# Online piety and its discontent: revisiting Islamic anxieties on Indonesian social media

**DOI:** 10.1080/13639811.2018.1415056

**Published:** 2018-02-22

**Authors:** Fatimah Husein, Martin Slama

**Affiliations:** ^a^ State Islamic University Sunan Kalijaga Yogyakarta, Yogyakarta, Indonesia; ^b^ Austrian Academy of Sciences, Institute for Social Anthropology, Vienna, Austria

**Keywords:** Indonesia, Islam, piety, *riyā’*, social media

## Abstract

In today’s digital age, many Indonesian Muslims utilise social media, such as Facebook, WhatsApp and BlackBerry Messenger (BBM), to express their piety. However, their religious online practices are not devoid of ambiguities, discontents and tensions. The article focuses on these specific consequences of being digitally pious in Indonesia. It examines how *riyā’*, an established concept in Islamic theology that refers to showing off one’s piety, has gained new relevance in the context of contemporary uses of social media for religious purposes. The article particularly discusses online Qur’an reading groups (ODOJ) and *sedekah* (charity) activities that utilise social media, and asks how Muslims deal with the problem of *riyā’*, which is strongly discouraged in Islamic theology, and with the discontent and anxieties it generates. At the same time, it reveals that the responses to the challenge that *riyā’* poses vary greatly and that Indonesian Muslims have found different ways to overcome it.

## Introduction

This article presents findings of a larger project that attempts to explore Islamic religiosities from the angle of everyday uses of social media in Indonesia.^1^ It revisits a concept called *riyā’* in Islamic thought, that is widely discussed today in the context of Indonesian online piety. It is defined by the respected Indonesian Islamic scholar Quraish Shihab as ‘the act of showing off our ‘*ibadah* [worship of God] with the hope to be praised by others before, during, or after conducting that activity’ (Shihab 1996: 677). *Riyā’* (Ar. riyā') is a subject that frequently appears in classic Islamic literature, especially in Islamic mysticism (*tasawwuf*). The great Sufi master Abū ‘Abdullāh al-Muḥāsibī (d.857 CE) emphasised that Muslims should know *riyā*’ so that they can avoid it and Allah will accept their prayers. In his *Adab an-Nufus* he mentions important techniques for how to avoid *riyā*’, such as introspection (*muḥāsaba*), evaluation of one’s deeds (*murāja’a*), and taking caution (*murāqaba*) (al-Muḥāsibī 1991: 42, 60, 95).

Many Muslims believe that their good deeds will be recognised by Allah as ‘*ibadah* and that these deeds could save them on Judgment Day. However, al-Muḥāsibī has warned that these good deeds could, on the contrary, lead them to hell if they are performed with *riyā*’ in their hearts. Moreover, Muslims who do something good in order to be praised by others would not only receive punishment later in the hereafter but would already feel discontent in this world. According to al-Muḥāsibī (1991: 57, 73–4), a sign of *riyā*’ is being happy when one is praised so that one loves one’s physical ‘*ibadah* more than its spiritual benefits. Another sign is that people who commit *riyā*’ would also hate it to be criticised.

Similar to al-Muḥāsibī, perhaps the most influential medieval Islamic scholar Abū Ḥāmid al-Ghāzalī (d.1111 CE, henceforth the Indonesian transliteration of his name will be used) clearly stated that *riyā*’ is unlawful (*haram*) and that Allah hates those who have *riyā*’ in their heart when performing ‘*ibadah* (al-Ghāzalī 2008: 360–6).^2^ Referring to the Qur’an – especially 107: 4–6, ‘So woe to the worshippers, who are neglectful of their prayers, those who (want but) to be seen (by men)’^3^ – and Hadith, al-Ghāzalī put a heavy emphasis on the dangers of *riyā*’ that he perceived as destroying ‘*ibadah.* He also identified *riyā*’ as a form of *shirk* (polytheism), since one is actually not worshipping God but others when performing prayers without exclusively having God in mind. One could also speak here of the problem of directionality that accompanies one’s pious deeds that can only be counted as pious if they are directed towards God only and nothing else. Furthermore, he underlined that those who commit *riyā*’ would not receive any rewards from Allah in the hereafter and they would even face severe punishment by being placed in hell.

Interestingly, Quraish Shihab (1996: 661) discusses *riyā*’ within the context of jihad which he defines as one of Allah’s ways to test human beings, since he understands jihad as associated with patience and perseverance. By quoting a saying of the Prophet Muhammad, he underlines the importance of struggling (jihad) against our carnal desires that Satan likes to whisper in our heart, including *riyā*’ which has to be avoided, since Allah would not accept any ‘*ibadah* that comprises an element of *riyā*’ (Shihab 1996: 677).

Avoiding *riyā*’ is perceived by both classical and contemporary Islamic scholars as crucial when worshipping God; *riyā*’ is seen as substantially devaluing prayers and can have serious consequences ranging from God’s rejection of one’s ‘*ibadah* to severe punishments, including the torments of hell. Since *riyā*’ is about showing off, about displaying one’s piety, the visibility of Islamic practice becomes key for understanding the debates about *riyā*’. Moreover, in today’s era of the continuing rise of social media, the visible becomes increasingly mediated. Current worries and anxieties about *riyā*’ in Indonesia thus can be seen as an example of how Muslims deal with ‘the implications when religion appears online’ (Campbell 2005: 1), i.e. when not only Islamic practice becomes mediated but mediation also becomes part of religious practice and thus constitutive of piety (see e.g. Eisenlohr 2009; Engelke 2010; Hirschkind 2011).

According to Campbell (2013: 1), this has far-reaching consequences, since ‘digital religion’, being present on a variety of online media ranging from websites to mobile apps, ‘does not simply refer to religion as it is performed and articulated online, but points to how digital media and spaces are shaping and being shaped by religious practice’. Various studies have shown that in these media spaces, practices – whether or not associated with a particular religion – address pressing ethical issues and invite moral judgements (Gershon 2010; Madianou 2014; Miller 2016). In such contexts, as Barendregt (2009: 83) has observed in Indonesia, communication technology experiences a ‘domestication’ within Islamic circles generating ever ‘more eye-catching examples of Islamic mobile religiosity’. Likewise, mobile and digital religion in Indonesia today cannot be separated from broader religious patterns of consumption that have emerged in the last decades with the rise of a Muslim middle class. As Fealy and White (2008: 2–3) have argued, Indonesian Muslims express and communicate their faith, beyond the traditional five pillars of faith, in various ways: they wear ‘traditional Islamic dress, buy only *halal* products, put their money in sharia bank accounts, log on to Islamic websites, observe the voluntary prayers and engage in charitable work for Islamic foundations’. This list can be extended by additional practices such as watching television broadcasts of Islamic preachers, undertaking pilgrimages to the burial sites of Islamic saints, and buying Islamic art that is displayed in homes and workplaces; and most importantly, today Indonesian Muslims like to present these practices on their social media accounts.

What we want to emphasise in this regard is that current Islamic online expressions constitute a preliminary outcome of the entanglements of technology and religion in Indonesia that started in Suharto’s New Order period (1966–1998). New Order ideology was eager to emphasise that technological advancement and spiritual well-being were compatible (Amir 2009), which had effects on the rise of the internet in the late New Order and the post-Suharto eras when the available communication technologies were used by a variety of (religious) actors (Hill and Sen 2005; Lim 2006). The contemporary trend of employing social media for enhancing one’s piety thus can be seen as a continuation of earlier developments, although some of today’s popular practices are unprecedented due to technological change and are part of the digitisation of various Indonesian spheres of life (Jurriens and Tapsell 2017). However, at the same time, the anxieties that are associated with these practices are not new, and this particularly applies to the discontent that many Muslims feel today with regard to *riyā*’.

This discontent is strongly connected to new online practices that make visible the everyday life of Indonesian Muslims, including their religious life. In the context of social media, being religious online always also means presenting one’s piety to an audience and engaging with a spectatorship that is by far larger than the one pious Muslims usually encounter in their offline environments. In other words, online religiosity bears a greater risk of *riyā*’ or of being accused of *riyā*’ than offline religiosity, or at least, it comprises new potentials for committing *riyā*’. The next sections take a closer look at how Indonesian Muslims deal with this risk in the context of two pious online practices: participating in online Qur’an reading groups and being active in Islamic charities.

## Online Qur’an reading groups and the problem of *riyā*’

In recent years, committing oneself to read one section (*juz*) of the Qur’an everyday has become popular in Indonesia . This practice is called ODOJ, standing for One Day One Juz (see also Nisa 2018, in this special issue; and Slama 2017b). There is a national Qur’an reading movement that bears this name, but ODOJ is also organised by Islamic preachers and by *majelis taklim* (religious study groups) that are mainly run by women. Despite this diversity, the reporting system that is used by the different groups is very similar, with WhatsApp being the most popular messaging app on which the Qur’an readers report to their group. Since the Qur’an has 30 sections, the online group of 30 people is expected to finish reading the whole Qur’an every day given that each member of the group has read the section that was assigned to her or him and has reported her or his reading activities before a particular deadline in the evening. Disciplined Qur’an readers will thus have read the whole Qur’an within a month, after which a new circle starts. In mid-2014, one of the authors of this article, Martin Slama, was allowed to join an ODOJ WhatsApp group as an observer (and is still a member of the group). The WhatsApp group is run by an Islamic preacher from Solo, Central Java, who lives in Jakarta. He started with one WhatsApp group, which rose to 13 groups, and from early 2015 onwards Fatimah Husein participated not only as an observer but also as an active Qur’an reader in one of his groups. The preacher, Ustadz Husein Nabil Assegaf, stems from a well-known family of Islamic scholars of Hadhrami Arab descent. Before the rise of new media, Ustadz Nabil who is following the footsteps of his father, the late Ustadz Najib Assegaf, a well known preacher from Solo, used to convey his *dakwah* (proselytisation) in various offline religious settings, but today he is going increasingly online using Facebook, Twitter, WhatsApp etc.^4^

Ustadz Nabil’s Qur’an reading programme, also known as *Tadarus al-Qur’an* (Reading the Qur’an) has very clear rules. Beginners receive the following information: At 8 pm every night the group coordinator, a person close to Ustadz Nabil or one of his family members, assigns a certain section of the Qur’an to each WhatsApp group member, and they have to report within 24 hours whether they have finished reading their section or whether they were not able to do so. The only acceptable excuses for not reading it are when female members have their period, during 40 to 60 days after giving birth, and serious illness (most members of the Qur’an reading groups are women). Not reporting to the coordinator within the 24 hours is counted as violation of the rules. Violating the rules three times results in being excluded from the group, which can only be re-joined in the next month. Every two months there is a ‘night of mercy’, however, in which all the members who have violated the regulations are pardoned and their violation records are cleared.

Discipline, consistency (*istiqomah*), and supporting each other characterise this Qur’an reading activity. For example, when a group member is unable to complete the assigned section, other members will take over her obligation to ensure that the group has completed reading the whole Qur’an on that day. Initially, members of the groups were very enthusiastic in taking the unread sections. However, this changed with the establishment of a separate WhatsApp group for volunteers comprising Qur’an readers from all of Ustadz Nabil’s groups. Even though easier coordination was behind the introduction of this new group, it simultaneously prevented these volunteers from committing or from being seen as committing *riyā*’ – in this case, showing to the other group members that they could read more sections of the Qur’an. Indeed, some people think along this line, i.e. that joining ODOJ through a social media app such as WhatsApp can easily entrap one into *riyā*’. For example, a relative of Fatimah Husein approached her because she also wanted to join the ODOJ group. Yet she soon changed her mind, as she came to the conclusion that it is better to read the Qur’an individually at home. She considered joining ODOJ through social media as prone to *riyā*’.^5^ This hesitation echoes al-Ghāzalī's (2008: 361) concern that it is important to avoid deeds that might lead one to commit *riyā*’ and thus save one from the torments of hell. Yet this is only one possible way to deal with the problem of *riyā*’ today, as the following example indicates.

Three years after her parents had passed away, Secha Ali, another woman from Solo’s Hadhrami community, decided to join an online ODOJ group (organised by a female Islamic preacher). She felt that this activity created serenity in her heart and she considers ODOJ as a good way to discipline herself by reading the Qur’an daily. However, when she shared her positive experience with her friends, she was asked whether this activity could be considered as *riyā*’. Her friends reminded her that by having joined ODOJ many people know that she reads one *juz* per day, as with the other members of her ODOJ group, and those who attend their monthly offline meetings (called *kopdar* from *kopi darat*, literally ‘having a coffee together on earth’). In fact, Secha Ali is aware of the danger of *riyā*’ in these activities, but she argued that the main objective of joining the Qur’an reading group is not to show off to other people. According to her, it does not matter if someone unintentionally commits *riyā*’, as long as she has good intentions in her heart.^6^ Whereas Secha Ali acknowledges the danger of *riyā*’, as the above mentioned theologians warned of, it did not deter her from joining this community of Qur’an readers, since she is convinced that what matters most are her genuine intentions.

Interestingly, there are also other ways to organise Qur’an reading groups that can solve the tension between pious online expressions and the risk of committing *riyā*’. For example, Ustadzah Ulya, a woman of Hadhrami descent living in Solo, uses a simple form for reporting the Qur’an reading in her ODOJ groups. At the beginning, she approached committed Muslim women in Solo’s Hadhrami community who can read the Qur’an fluently. Today, she runs seven groups with female members only, and communicates with her members mostly through text messages (Short Message Services, SMS), and infrequently via BlackBerry Messenger (BBM). She argued that many of her ODOJ group members are not tech-savvy people and they cannot afford more sophisticated smartphones. Thus, each member is given a simple form consisting of 30 blank columns for the *juz* (section) with 12 rows referring to the months. When someone becomes a member of a group and is assigned with *juz* 10, for example, she then writes *juz* 10 in her first column and ends with *juz* 9 at the end of the month. Ustadzah Ulya argues that this simple paper form has the side benefit that the members have a written record of their reading cycles. With the growing number of members, there is now a WhatsApp group that all members are allowed to join (if they own a smartphone). However, this WhatsApp group is only used to facilitate easier communication, and not for reporting the Qur’an reading.^7^ As a consequence, *riyā*’ cannot take place in this online setting.

Unlike the general trend that one can observe in Indonesia today where many members of offline prayer meetings are increasingly going online (Slama 2017a), this ‘manual’ ODOJ initiative is consistently going offline. The women also meet once a month offline, marking the completion of reading the thirtieth *juz*, when they use this opportunity for socialising and for discussing religious issues from a female perspective. As with the ODOJ groups of Ustadz Nabil, the followers of Ustadzah Ulya are also required to complete the reading within 24 hours by *maghrib* (the end of the day) but there is no need to report to the group coordinator, except for the unread sections. This means that the members only report when they were not successful, displaying their lack of piety, so to say, which can of course not be interpreted as *riyā*’. Moreover, Ustadzah Ulya has good connections to a *pesantren* (Islamic boarding school) in Solo, where she can easily find volunteers to complete the unread sections. This system has proven to attract more new members who are assured that someone else will take over reading the sections assigned to them, when they are unable to do so.

However, most ODOJ groups in Indonesia follow the system that their members have to report back on whether they were successful in reading their section of the Qur’an or not. So what is the opinion of the preachers that run such ODOJ groups with regard to *riyā*’? Ustadz Husein Nabil Assegaf’s stance on the issue is a good example in this regard, since we frequently encountered the argument put forward by him (or variations of it) during our research. According to him, *riyā*’ depends on one’s intention (*niat*) when worshipping God, and only God can know the real intention of a particular person. Thus, *riyā*’, he emphasises, is ‘a matter between you and God’.^8^ This argument turns the public performance of worship into a matter that cannot be discerned – and thus judged – by others and underlines the responsibility of every Muslim to investigate the intention that lies behind one’s pious deeds.^9^ In other words, avoiding *riyā*’ demands a high degree of awareness and self-discipline when worshipping God.

Furthermore, the emphasis on *niat* can be read as an attempt to accommodate contemporary technological and social transformations, of which the appropriation of digital communication in everyday life is an important aspect. Thus, instead of being extra cautious regarding the matter of *riyā*’ as prescribed by the classical scholars al-Ghāzalī and al-Muḥāsibī, Ustadz Nabil suggests leaving it to the divine judgment of one’s intentions. At the same time, he nevertheless positions himself within this tradition of scholarship, since emphasising *niat* implies paying heed to whom one’s pious deeds are directed. And if they are directed towards God alone, one has not committed *riyā*’ as a form of *shirk* (polytheism) as al-Ghāzalī has warned of in his classic book.

As we have outlined in the introduction, *riyā*’ is a problem that has become a subject of the treatises of Islamic scholars, mediaeval and contemporary, attempting to make Muslims aware of its dangers. It has also become a subject of discussion among participants of *majelis taklim*, particularly those who run online Qur’an reading groups and are very active on various social media. One of the most active groups in Yogyakarta is the Majelis Sahabat Cinta (the *majelis* of the Friends of Love) that also invites preachers from outside the city, such as Ustadz Nabil. Among its many activities, the group issues so-called pocket books comprising ‘religious advice’ (*nasehat-nasehat agama*). In the first booklet of this series, the second chapter is dedicated to *riyā*’, entitled ‘Avoiding *riyā*’’ (*Menghindari riyā’*). In two pages, it explains the different forms of *riyā*’ and how difficult it sometimes is to realise that one is actually committing *riyā*’. Given the various online and offline activities in which *majelis taklim* are involved today, it does not come as a surprise that *riyā*’ has emerged as an issue discussed by women that regularly attend these Islamic study groups. This is also supported by the fact that at least some of the preachers who teach at the *majelis* are graduates of Islamic boarding schools where classical Islamic texts, notably *Iḥyā’ ‘Ulūm al-Dīn*, are taught. While Qur’an reading is one of their activities, actively supporting Islamic charities is another.

## The pitfalls of online *sedekah*

The second online practice where discussions of *riyā*’ has become particularly apparent in Indonesia today concerns *sedekah* activities, charitable donations that are voluntary in contrast to the mandatory *zakat*. Islamic charities have also gone increasingly online, generating further challenges for those who run them as well as for those who like to engage in *sedekah. Sedekah* is considered a noble act in Islam as reflected in the two Islamic foundational texts, the Qur’an and Hadith. In the Qur’an (57: 18), for example, Allah says: ‘For those who give in Charity, men and women, and loan to Allah a Beautiful Loan, it shall be increased manifold (to their credit), and they shall have (besides) a liberal reward.’ This is supported by a Hadith stating: ‘The upper hand that gives is better than the lower hand that takes.’^10^ And there are several other Hadith that remind Muslims of the principles of giving to others. One popular Hadith states that among those who would receive protection from Allah on the Day of Judgment is ‘a person who gives charity and conceals it (to such an extent) that his left hand does not know what his right hand has given.’^11^ This saying of the Prophet Muhammad clearly refers to the problem when donating becomes a public act, when other people can observe one’s *sedekah* activities, reminding Muslims that even they themselves should not fully perceive their donations, let alone that other people should know.

In Indonesia in general, and in Yogyakarta in particular, where most of the research for this section was conducted, *sedekah* initiatives have become manifest in various ways: helping the victims of natural disasters, distributing lunch boxes to orphanages, facilitating access to hospitals for those who are sick and poor, and even buying land for building an orphanage where the children are taught to memorise the Qur’an. Many Islamic charities in Yogyakarta today use social media, such as WhatsApp, Instagram, Facebook, and Twitter, that have become essential for their fund-raising activities. Social media are employed to effectively connect ‘*sedekaholics*’ or ‘*sedekahlovers*’ (popular terms used to refer to people who regularly donate) with online *sedekah* organisers.

Rumah Hati Jogja (RHJ), a charity founded in early 2011 after the eruption of Mount Merapi in Yogyakarta, for example, has been quite successful in establishing networks through Facebook, WhatsApp and BBM. They directly go to the disaster locations and distribute aid with the help of local people. RHJ purely depends on social media, especially Facebook, to collect donations. Their yearly turnover is about seven billion rupiah (about US$520,000). Moreover, Ustadz Rully Arta Nugraha, the founder of RHJ, is active in sending his *dakwah* messages through WhatsApp, and he regularly uploads information about the charity activities of his organisation on Facebook.

Some of his followers join him in his social media activities, such as Sri Yanti who also regularly attends *majelis taklim* meetings. During one of the meetings, Sri Yanti learnt that one of her friends had cooked food for RHJ that was delivered to the needy. They then donated money for delivering more food boxes on the following days but soon realised that this method was ineffective. Trying to find a solution for the problem of regularly receiving sufficient funds to uphold their activity, they considered posting an invitation to donate on their WhatsApp Display Pictures that could be seen and read by all the people on their contact lists. However, they were uncertain if this idea could entrap them into *riyā*’. They then contacted Ustadz Rully Arta Nugraha to confirm this and they were told that *riyā*’ goes back to one’s original intention (*niat*) that will be judged by Allah and not by other people. Ustadz Rully thus gave basically the same answer as Ustadz Nabil did in the context of Qur’an reading groups. In fact, this approach of using social media to call for donations has proven to be effective and has motivated Muslims from Sri Yanti’s and her friends’ contact lists to donate money, including Muslims from other countries such as Iraq and Kuwait.^12^

In an effort to expand his activities, Ustadz Rully created Waroeng Santri, an online grocery and fresh food shop managed via a BBM group. The shop mainly sells rice and cooked food and regularly uploads information of the menu, price, and how to order via BBM. Users are also informed that 10% of the total expenses is allocated for the orphans that live in the *pesantren* established by RHJ. Moreover, sometimes this BBM group is also used by RHJ to call for donations for its various activities. The following messages exemplify this practice:

Those who believe in God’s help are extraordinary people and they are not like ordinary people. Let us donate for our programme of the blessing month of Ramadhan.

That our wealth is halal is one of the keys to save us from punishment in the grave.

This is our chance to trade with Allah, so let us donate our wealth (*jihad harta*) in our attempt to spread Islam.^13^

These quotes indicate that it is apparently not seen as problematic to urge people online to donate, i.e. to make an announcement on social media platforms that one is organising *sedekah* activities. However, the problem of *riyā*’ comes up when one announces that one has actually donated and, in doing so, discloses one’s identity as a donor. Then, according to Ustadz Rully, it is important to investigate one’s intention why one has donated and made that public.^14^

The year 2011 witnessed the onset of another *sedekah* organisation that is well known in Yogyakarta today. It was founded by the businessman Saptuari Sugiharto who felt the need to help orphanages and the poor through Sedekah Rombongan (*rombongan* refers here to donating together in a group). He likes to motivate people to donate through his online articles, first through his blog, and later via Twitter and Facebook. As he explained, once people read his real stories about the suffering of orphans, of people with disability, and of those who are very ill, they would feel empathy and start to donate. Today, the monthly turnover of Sedekah Rombongan is around 1 billion rupiah (US$75,000). Interestingly, many of those who donate money to Sedekah Rombongan do so anonymously. While realising that these people did so in their attempt to avoid *riyā*’, Saptuari argued that *riyā*’ is a matter of the heart and there is no machine that could measure the degree of *riyā*’ in our heart.^15^ He also referred to *sedekaholics* that give their testimonies related to their *sedekah* via Twitter, Facebook and WhatsApp groups:

Brother Saptuari, I just want to share. At the end of May I transferred a certain amount of money to Sedekah Rombongan via BCA [Bank Central Asia, one of Indonesia’s big banks], and three days later I received cash 10 times more [than the amount of the donation]. Really 10 times bigger.

I donated to Sedekah Rombongan on Sunday, and I received a free ticket for *umroh* [pilgrimage to Mecca outside of the the main pilgrimage month] on Tuesday the next week.

Brother Saptuari, I donated 270 thousand rupiah, and I received back 2.7 million. Then I donated 2.7 million and received back 28 million. I donated again, 6.5 million and received back 100 million. How should one call this, brother? I am at a loss for words.^16^

It is interesting to note that many of the *sedekaholics* connect their donations to ‘instant’ material rewards from Allah thereby at least implicitly alluding to the scripture mentioned above. At the same time, their testimonies embody ambivalence. While sharing one’s experience online might motivate others to actively engage in *sedekah*, these testimonies could easily entrap them into *riyā*’, since some clearly exhibit detailed information of their respective donations. Moreover, since there is no machine that can ‘measure’ the degree of *riyā*’ in one’s heart, as Saptuari asserted, his followers are left alone to decide whether their online behaviour is an expression of *riyā*’ or not.

Similar to Sedekah Rombongan, another charity organisation in Yogyakarta, Simpul Sedekah, uses social media extensively to solicit funds and to report its activities. However, since its establishment in late 2013, its social media uses have generated different responses. Some people share the spirit of this organisation and financially support it, but others question it and argue that one’s charity activities should not be seen by others, and should also not be ‘announced’ on social media. Haris Hermawan, who established this organisation, responds to this critique by stating that uploading information on Facebook or sharing it via WhatsApp is not meant for *riyā*’, which, he nevertheless underlined, should be avoided in *sedekah* activities, but it is meant to call other Muslims to join these charity actions. He further argued that the Qur’an emphasises the importance of *sedekah*. From his point of view, as the coordinator (or collector) of *sedekah*, what is more important is to be accountable; and social media are very useful for this aspect of transparency, in showing the amounts donated and the uses put to the donations. Moreover, he asserted, they can be used to openly invite people to donate.^17^ The following are examples for the calls to donate that he likes to post on his Facebook account:

The key in getting an easy life is through *sedekah*. When you help others with your wealth, your life will be eased [by Allah].^18^

There are many of our brothers and sisters who are less fortunate. Let us increase our gratitude with *sedekah*.^19^

Besides giving food boxes to various orphanages and conducting programmes for the needy called Breakfast on the Street and *Sahur* on the Street,^20^ Simpul Sedekah is also active in helping the poor to receive proper treatment in hospitals. Information about these activities is also uploaded on Haris’ Facebook account and WhatsApp groups. In addition, the organisation likes to engage in rather unique initiatives, such as *Sedekah Tiket* and *Ngaji Satu Juz, Gratis Satu Porsi*. *Sedekah Tiket* was an online call to buy movie tickets for orphans to watch the film *Surga menanti* (Heaven is waiting), which tells the story of a young boy who memorises the Qur’an. Simpul Sedekah, in collaboration with other charity organisations in Yogyakarta including Majelis Mutia Sholeha, ODOJ Yogya, and Bahagia Bersama Qur’an (BBQ) that have online platforms, was able to raise enough funds for 150 orphans who could then watch the movie. Before watching it, the children read one *juz* of the Qur’an and participated in a session sponsored by the ASEAN Economic Community where they were taught about the importance of learning various skills, including languages as well as having ‘big dreams’. The second initiative heavily advertised on social media was *Ngaji Satu Juz, Gratis Satu Porsi* where Haris invited people to come to his restaurant and read one section of the Qur’an for a free meal.

Our field research in the community of *sedekah* donors has brought us to Achmad Hasan and his wife Nestri. Their friends label them ‘the king and queen of *sedekah*’ (*raja dan ratu sedekah*) explaining that the couple does not donate their money because they are rich but rather that their keenness to donate has resulted in their getting richer. Achmad Hasan, who owns several restaurants and apartments for rent, loves to ride large motorcycles such as Harley Davidsons, and believes that the wealth that will accompany us when we pass away is that which we have donated. Achmad and Nestri regularly donate to numerous *sedekah* organisations and have a huge kitchen in their house where they prepare lunch and dinner boxes each Monday and Thursday that are then delivered through Simpul Sedekah. They are not members of any of these organisation, but consider themselves ‘contributors’. For their charity activities, they use BBM, WhatsApp, Path, and Facebook. They argue that these social media help them in communicating with their religious teachers and in supporting their religious activities, including *sedekah*. However, Achmad is aware of the risk of *riyā*’ in his *sedekah* activities and argues that only the Prophet Muhammad does not have *riyā*’ in his ‘*ibadah*. Other people, including him, have it in their heart but need to fight against it by always reciting *istighfar* (asking forgiveness from Allah).^21^

We read Achmad’s statement about *riyā*’ against the backdrop of the continuing status competition among Indonesian middle-class Muslims. Social media are certainly not devoid of these dynamics as they are perfect platforms for visibly asserting one’s middle-class status and boosting the ways in which middle-class Muslims can present themselves to their semi-public online communities. Hence, while social media provide the unrivalled platforms, *sedekah* appears to be the ideal way to practise one’s religion by enhancing one’s social status and, according to the accounts above, even one’s financial situation. Therefore, it is not surprising that Achmad holds the opinion that *riyā*’ is actually never absent from *sedekah* and that the only thing that he and others, meaning Indonesian middle-class Muslims, can do against it is fighting it and asking God for forgiveness.

The above examples on *sedekah* confirm that the approaches of our interlocutors and those of theologians, especially al-Ghāzalī, with regard to *riyā*’ can considerably differ. For al-Ghāzalī (2008: 361–2) it is very important to be particularly cautious in the act of giving to others, since *riyā*’ could easily come along with it. He repeatedly warns of the serious danger of *riyā*’ equating it to *shirk* (polytheism), and stated that those who commit *riyā*’ will be placed in hell. However, our interlocutors chose to emphasise the importance of performing good deeds, including *sedekah*, even though they realise the danger of *riyā*’ when they are going online. They feel *riyā*’ constitutes a problem that cannot be completely avoided but can be dealt with by engaging in other pious practices such as prayer. Moreover, among those who are involved in *sedekah* activities the interpretation that *riyā*’ cannot be discerned by others and will only be known by God seems to be widespread.

## Conclusion

As we have indicated throughout this article,*riyā*’ is not a new topic and has been discussed by prominent Islamic scholars many centuries ago. However, as we have also attempted to show, it has gained new significance in today’s social media age when a kind of revival of discussions about *riyā*’ seems to be taking place (Husein 2017). Social media provide numerous opportunities for Indonesian Muslims to express their piety online and practise their religion in mediated forms. Yet precisely in these expressions and practices and their digital traces, that are hard to undo once they have appeared online, lie considerable risks and challenges, since every attempt to worship God can be redirected towards other goals, such as impressing others which would constitute an act of *riyā*’. When becoming aware of these risks related to *riyā*’ and receiving warnings about it from others, especially from preachers, Indonesian Muslims show a variety of responses. In the case of online Qur’an reading groups, some Muslims indeed refrain from taking part in these groups or resign once they have realised the danger of *riyā*’, whereas others do not see a problem at all in reporting their pious practice on messaging apps. There are also interesting attempts by the organisers of these groups to deal with *riyā*’. While some preachers define it as an issue that Muslims can only solve in their personal relationship with God, assigning it to a realm that is incomprehensible for a third party, others organise the Qur’an reading in a way that attempts to protect the readers from committing *riyā*’ by introducing a complex mix of offline and online reporting. Or they alter online communication to the extent that committing *riyā*’ becomes almost impossible from the start, such as obliging members to report only when they were unable to read their sections.

In the case of *sedekah* activities, the responses to *riyā*’ seem to be even more varied. These activities are made visible in many ways and by people occupying different positions in the *sedekah* economy. Those who run the charities depend to a great extent on social media and thus defend their postings as attempts to motivate people to donate and generally emphasise the dominant interpretation of *riyā*’ as a matter of the heart that only God knows and no other human being can judge or ‘measure’. On the side of the donors, however, we encounter more caution, since some of them avoid disclosing their identity. However, we can also observe the exact opposite with people posting under their real name and exactly how much they have donated and how much they ‘received back’ from God. In addition to that and in comparison with the positions of the classical scholars al-Ghāzalī and al-Muḥāsibī, we have encountered the perhaps most unorthodox understanding of *riyā*’ that nevertheless quite accurately reflects the middle-class environment in which most *sedekah* activities take place in Indonesia, namely that *riyā*’ is unavoidable but can be pardoned by God if one asks Him for forgiveness.

This article is aimed at unveiling the variety of practices and interpretations that have emerged with the introduction of social media in the religious lives of many Indonesian Muslims. While social media allowed new ways of expressing piety and expanded possibilities of Islamic sociality and organisation, they also generated ambivalence and anxiety. However, this latter aspect of discontent that became manifest through the contemporary revival of discussions of the concept of *riyā*’ is not met with a uniform response. Instead, Indonesian Muslims renegotiate *riyā*’ in various ways that have different consequences for their religious practices. Yet, staying completely offline due to the dangers of *riyā*’ or restricting one’s online activities to purely non-religious issues seems to be an option that only very few Muslims choose, reflecting the degree with which social media have already penetrated the religious lives of many Indonesians.

## Footnotes

1 See the funding information at the end of this article. For this article, offline research was mainly undertaken in Yogyakarta, Solo, and Jakarta by the authors.

2 Al-Ghāzalī is taught in many Indonesian Islamic educational institutions, such as Islamic boarding schools (*pesantren*) associated with Indonesia’s biggest Islamic organisation Nahdlatul Ulama where his book *Iḥyā’ ‘Ulūm al-Dīn* is widely read (van Bruinessen 1994).

3 All quotations from the Qur’an are taken from Abdullah Yusuf Ali, *The Holy Qur’an*, <www.theonlyquran.com>, accessed 7 August 2017.

4 Fatimah Husein is of Hadhrami Arab descent herself and Martin Slama has conducted several research projects about Indonesia’s Hadhrami community; thus they had the privilege of easily connecting with Hadhrami *ustadz* (male preacher) and *ustadzah* (female preacher) online and offline, and to carry out interviews with them and their followers. Arabs from the Hadhramaut, a region located in the eastern part of today’s Republic of Yemen, have migrated to Southeast Asia for centuries. Today, they form distinct communities in Indonesia and are well established especially in Indonesia’s Islamic field. Indonesians of Hadhrami descent are found among the country’s most popular Islamic preachers (Abaza 2004; Alatas 2011; Nisa 2012; Slama 2011).

5 BlackBerry Messenger (BBM) chat, 22 October 2014.

6 Interview with Secha Ali in Solo, 24 October 2014.

7 Interview with Ulya in Solo, 2 May 2015.

8 Conversation with Ustadz Husein Nabil Assegaf in Jakarta, 7 February 2015.

9 It is tempting to frame this approach to *riyā*’ by resorting to the public/private distinction. However, in this context it is more appropriate to refer to the Islamic concepts of *zāḥir* (the manifest) and *bāṭin* (the hidden or unseen), known in Indonesia as *lahir* and *batin*, that imply a different notion of the self and interiority in relation to the seen and unseen worlds than the post-Enlightenment differentiation of public and private spheres.

10 Muslim (2004: 400, Hadith number 1033).

11 Muslim (2004: 399, Hadith number 1031).

12 Interview with Ustadz Rully Arta Nugraha, 30 January 2015.

13 These messages were recorded by Fatimah Husein in May 2015.

14 Interview with Ustadz Rully Arta Nugraha, 30 January 2015.

15 Interview with Saptuari Sugiharto in Yogyakarta, 1 March 2016.

16 Private documentation by Saptuari Sugiharto that is also displayed at the walls of his office.

17 Interview with Haris Hermawan in Yogyakarta, 10 February 2016.

18 Haris Hermawan’s Facebook account, posted on 19 June 2017.

19 Haris Hermawan’s Facebook account, posted on 22 June 2017.

20
*Sahur* refers to the meal that Muslims have before sunrise during the fasting month.

21 Interview with Achmad Hasan and Nestri in Yogyakarta, 27 February 2016.
